# Comparative Analysis of Transcriptomic Changes including mRNA and microRNA Expression Induced by the Xenoestrogens Zearalenone and Bisphenol A in Human Ovarian Cells

**DOI:** 10.3390/toxins15020140

**Published:** 2023-02-09

**Authors:** Éva Márton, Alexandra Varga, András Penyige, Zsuzsanna Birkó, István Balogh, Bálint Nagy, Melinda Szilágyi

**Affiliations:** 1 Department of Human Genetics, Faculty of Medicine, University of Debrecen, H-4032 Debrecen, Hungary; 2 Doctoral School of Molecular Cell and Immune Biology, University of Debrecen, H-4032 Debrecen, Hungary; 3 Faculty of Pharmacy, University of Debrecen, H-4032 Debrecen, Hungary; 4 Division of Clinical Genetics, Department of Laboratory Medicine, Faculty of Medicine, University of Debrecen, H-4032 Debrecen, Hungary

**Keywords:** xenoestrogen, zearalenone, mycotoxin, bisphenol A, ovarian cancer, transcriptomics, microRNA, RNA sequencing

## Abstract

Xenoestrogens are natural or synthetic compounds that mimic the effect of endogenous estrogens and might cause cancer. We aimed to compare the global transcriptomic response to zearalenone (ZEA; mycotoxin) and bisphenol A (BPA; plastic additive) with the effect of physiological estradiol (E2) in the PEO1 human ovarian cell line by mRNA and microRNA sequencing. Estrogen exposure induced remarkable transcriptomic changes: 308, 288 and 63 genes were upregulated (log_2_FC > 1); 292, 260 and 45 genes were downregulated (log_2_FC < −1) in response to E2 (10 nM), ZEA (10 nM) and BPA (100 nM), respectively. Furthermore, the expression of 13, 11 and 10 miRNAs changed significantly (log_2_FC > 1, or log_2_FC < −1) after exposure to E2, ZEA and BPA, respectively. Functional enrichment analysis of the significantly differentially expressed genes and miRNAs revealed several pathways related to the regulation of cell proliferation and migration. The effect of E2 and ZEA was highly comparable: 407 genes were coregulated by these molecules. We could identify 83 genes that were regulated by all three treatments that might have a significant role in the estrogen response of ovarian cells. Furthermore, the downregulation of several miRNAs (miR-501-5p, let-7a-2-3p, miR-26a-2-3p, miR-197-5p and miR-582-3p) was confirmed by qPCR, which might support the proliferative effect of estrogens in ovarian cells.

## 1. Introduction

Estrogens (i.e., estrone, estradiol, estriol) consist of steroid hormones that coordinate the female reproductive system, as well as the development of secondary sexual characteristics. Furthermore, estrogens exert additional roles in the regulation of lipid metabolism, electrolyte balance, skeletal homeostasis, in the control of inflammation and in the coordination of the cardiovascular and central nervous system [1]. Due to their various functions, estrogen action might also be related to several pathological conditions including infertility, polycystic ovarian syndrome, endometriosis or to the development of gynecological cancers [2]. Estrogen response is primarily mediated by the contribution of intracellular nuclear receptors termed estrogen receptors (ER) that consist of ERα and ERβ encoded by the *ESR1* and *ESR2* genes. Due to the lipophilic nature of estrogen molecules, they can freely pass through the cell membrane and target ERs, which results in receptor dimerization and the translocation of the activated ER to the nucleus. As a consequence, ERs act as ligand-activated transcription factors that bind to genomic regions containing estrogen response elements (ERE sequences). That mechanism is known as direct genomic signaling. ER can also exert its effect by nondirect genomic signaling mediated by its interaction with various transcription factors such as Sp-1, NF-κB or AP-1 Fos/Jun dimers [1,3]. The nongenomic signaling of estrogens is mediated through G-protein-coupled ERs (GPER) that results in the rapid activation of AKT or MAPK pathways. Furthermore, ERs might induce a ligand independent signaling pathway that is activated via their phosphorylation by protein kinases, such as PKA, PKC or MAPK cascades [1,3]. It is well known that estrogen response might be also exerted by microRNAs (miRNAs) that are approximately 18–22 bp long noncoding regulatory RNA molecules. They act in post-transcriptional gene regulation via guiding the RISC complex to the 3′ UTR-region of target mRNAs that results in mRNA degradation or translational suppression [4]. The role of miRNAs in estrogen action might be exerted by several mechanisms: (i) ERα can interact with miRNA processing enzymes such as Drosha and Dicer; (ii) miRNAs might have an influence on the expression or activity of ERs (via targeting the transcripts of *ESR1/2* or their coregulators, such as SRC-1/NCOA1); and (iii) estrogen exposure causes altered miRNA expression [5,6,7,8].

The term xenoestrogens is referred to natural (produced by plants or fungi) or synthetic (e.g., industrial chemicals, medicinal drugs or body cosmetics) compounds that mimic the effect of endogenous estrogens [1]. Their effect is primarily based on their ability to bind to ERs (especially to ERα) or GPERs, that results in estrogen response. Furthermore, they are able to alter endogenous hormone signaling by the following mechanisms: (i) they might alter the expression of hormone receptors (e.g., estrogen, oxytocin or vasopressin receptors); (ii) they might induce or interfere with epigenetic mechanisms (e.g., by altering the expression of DNA methyltransferases or noncoding RNAs such as miRNAs); and (iii) they might alter hormone synthesis, transport or metabolism [9,10]. Due to their various effects on the endocrine system, these chemicals are regarded as endocrine disruptors that might cause several diseases including precocious puberty, infertility and cancer [9,11,12,13,14,15]. Bisphenol A (BPA) is one of the most hazardous chemicals in the group of synthetic xenoestrogens. BPA has been widely applied in the plastic industry since 1957 for the production of plastic materials such as hard plastics, epoxy resins, medical devices, dental sealants and the internal coating of food and beverage cans. BPA tends to leach from these plastic items, thus human exposure to BPA is considered to be high due to the consumption of food and drink stored in BPA-containing plastic containers, or to the inhalation of dust. As a consequence, BPA is readily detectable in human body fluids (e.g., in plasma, urine or in amniotic fluid) [12,16]. An example for natural xenoestrogens is zearalenone (ZEA), which is a mycotoxin (also called mycoestrogen) produced by *Fusarium* sp. (e.g., by *F. graminearum*, *F. oxysporum* or *F. culmorum*) that can often infect maize and cereal crops in the fields or during storage. ZEA possesses high stability, thus it remains stable during food processing, which might lead to the relatively high contamination of cereal products like bread, flour or malt. Additionally, humans might also be exposed to ZEA by consuming meat or milk products due to the fact that domestic animals are also exposed to mycotoxins by contaminated feed [17]. The maximum permissible limit of ZEA is 100–200 µg/kg in unprocessed cereals [18], which is frequently higher, especially in developing countries. Thus, ZEA contamination represents a relevant risk factor to human health [15,17].

Among women, ovarian cancer is considered to be the 8th most common cause of cancer death and it is the most lethal form of gynecological malignancy [19]. The role of estrogens in the development of ovarian cancer is suggested by the following observations: (i) estrogen has a proliferative effect to ER-positive ovarian tumor cells; (ii) the application of estrogen-based hormone replacement therapy, which is frequently used among postmenopausal women in order to decrease the symptoms of age-related diseases, represents an important risk factor for the development of ovarian cancer; (iii) a total of 81% of ovarian tumors express ER; and (iv) ovarian cancer cells might have the capability to synthesize estrogen [20,21,22]. However, little is known about the effect of xenoestrogens on the development of ovarian cancer. In our previous studies we have shown that ZEA and BPA induced cell proliferation and migration in the PEO1, ERα-expressing human epithelial ovarian cell line in physiologically relevant doses [23]. Furthermore, the exposure to estrogens induced significant transcriptional changes including the deregulation of estrogen responsive genes, as well as miRNAs [23,24,25]. Here, we aimed to study: (i) to monitor global transcriptomic changes in response to estrogen treatment in order to obtain a broad picture about the estrogen response of human ovarian cells; (ii) to identify key genes and miRNAs that are involved in this process; and (iii) to compare the effect of ZEA and BPA with physiological E2 in order to understand the effect of these xenoestrogens to human health. For this purpose, omics-based methods were applied including mRNA and miRNA sequencing. According to our results, ZEA and BPA induced characteristic transcriptomic changes that might promote tumorigenesis by supporting cell proliferation and migration. MRNA sequencing data suggest that the effect of ZEA was more comparable with E2 than BPA. Furthermore, we identified several miRNAs which expression was affected by estrogens.

## 2. Results

### 2.1. ZEA-Induced Changes in mRNA Expression Are More Comparable to That of E2 Than BPA

In our studies, we wanted to compare the effect of ZEA and BPA on the transcriptome to the response exerted by physiological E2 by mRNA sequencing. For this reason, the ERα-expressing, estrogen-sensitive PEO1 human epithelial ovarian cell line was applied [23]. According to our previous report, the tested estrogens including E2, ZEA and BPA induced cell proliferation and migration in this cell line in a dose-dependent manner [23]. First, we determined the optimal dose for the applied estrogens by quantifying the expression of the estrogen-sensitive *GREB1*, *CA12*, *DEPTOR* and *AGT* genes in PEO1 cells 8 h after exposure to 10 and 100 nM doses of E2, ZEA and BPA. These proved to be estrogen-sensitive genes in our previous studies [23]. According to our results, the addition of 10 nM E2 and ZEA resulted in a significant upregulation of the marker genes that were not increased significantly when the applied dose was elevated to 100 nM (Supplementary Materials Figure S1). However, the induction of *GREB1*, *CA12*, *DEPTOR* and *AGT* was not observed when BPA was applied in 10 nM concentration. They showed induction when the BPA was applied in 100 nM concentration and their expression level proved to be comparable to that of 10 nM E2 and ZEA (Supplementary Materials Figure S1). As a result, we used a 10 nM dose of E2 and ZEA and a 100 nM dose of BPA in our transcriptional studies. These observations are in good agreement with our previous studies [23].

**Figure 1:**
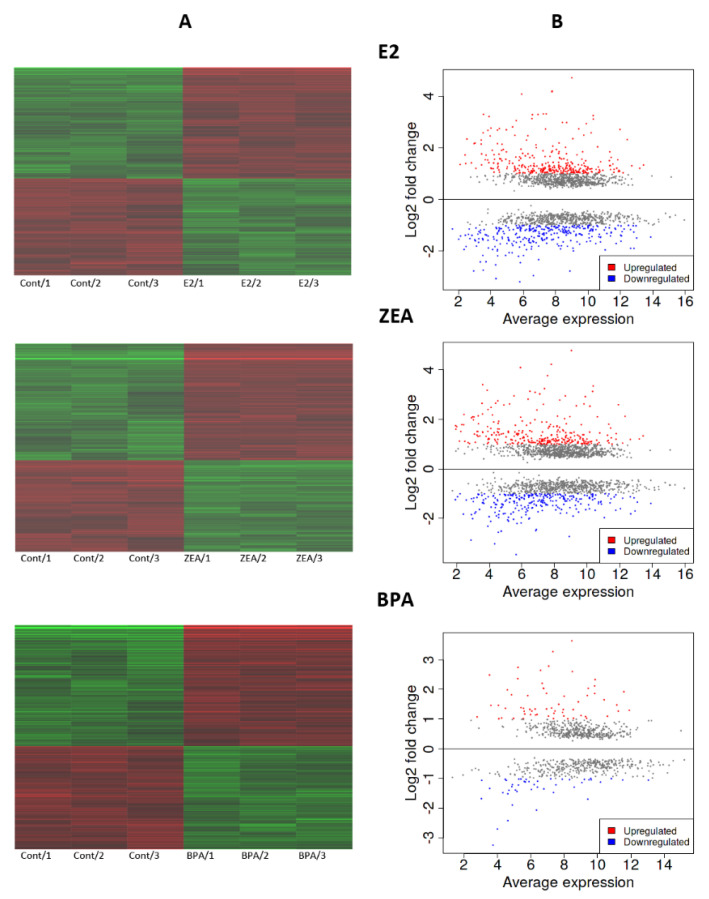
Gene expression alterations in response to E2, ZEA or BPA treatment. (**A**) Heatmap of genes that showed significant change in gene expression in response to estrogen treatment compared to the nontreated control (*p* < 0.05). Expression profile of the three replicas is presented (Cont: nontreated control). (**B**) MA-plot of up- and downregulated genes in response to estrogen treatment compared to the nontreated control. The extent of gene expression alterations is presented as Log_2_FC values. Log_2_FC > 1 means biologically relevant upregulation (red symbols); Log_2_FC < −1 means biologically relevant downregulation (blue symbols).

**Figure 2:**
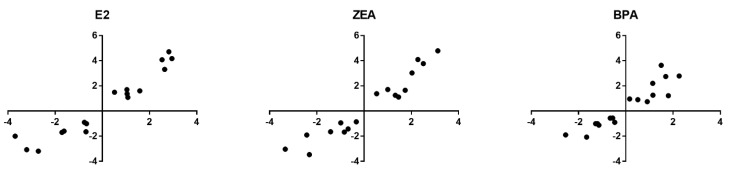
The validation of gene expression alterations by qPCR using 17 genes listed in Supplementary Materials Table S7. Gene expression alterations were determined as Log_2_FC values. *x* axis: Log_2_FC values obtained from qPCR. *y* axis: Log_2_FC values obtained from mRNA sequencing.

During the transcriptomics experiment, three replicates were also sequenced in the case of the nontreated control and treated samples. The effect of the estrogens was compared to the nontreated control culture. According to these data, all the tested estrogens were able to induce significant alterations in gene expression. In response to E2, ZEA and BPA exposure, we detected significant changes in the expression of 1847, 2019 and 901 genes, respectively (moderated *t*-test; *p* < 0.05; Figure 1A; Supplementary Materials Table S1). In order to characterize the extent of up- or downregulation, log_2_FC values were calculated using the iDEP.96 web tool applying the DESeq2 algorithm. We took into consideration changes that reach two-fold up- or downregulation that was considered to be the threshold of biological relevance. According to our analysis, 308, 288 and 63 significantly differently expressed genes (DEGs) was found to be upregulated (log_2_FC > 1) and 292, 260 and 45 DEGs were downregulated (log_2_FC < −1) in response to E2, ZEA and BPA, respectively (Figure 1B; Supplementary Materials Table S2). This suggests that the effect of ZEA was more comparable to that of E2, than to the effect of BPA. In order to validate these results, 17 genes were selected based on the degree of their expression change in order to assess the reliability of the mRNA sequencing experiment. Among the chosen DEGs, eight genes (*RBBP8NL*, *BLNK*, *TGMI*, *KRT4*, *BMF*, *CD24*, *NOTCH3* and *GBP3*) showed downregulation and nine genes (*MYC*, *EGR1*, *NOLC1*, *RRP12*, *MYBL1*, *GREB1*, *CA12*, *DEPTOR* and *AGT*) were upregulated with variable intensity according to the mRNA sequencing data (Supplementary Materials, Table S2). Validation was carried out by quantifying their expression by qPCR and the obtained expression values were compared with their expression level measured in the mRNA sequencing. The Log_2_FC values obtained with qPCR correlated well with the Log_2_FC values derived from the transcriptomic experiment (Figure 2). The Pearson correlation coefficient proved to be r = 0.95, r = 0.96 and r = 0.93 in the case of E2, ZEA and BPA treatments, respectively (Figure 2).

### 2.2. Transcriptomic Changes Induced by E2, ZEA and BPA Favored Cell Proliferation and Migration

**Figure 3:**
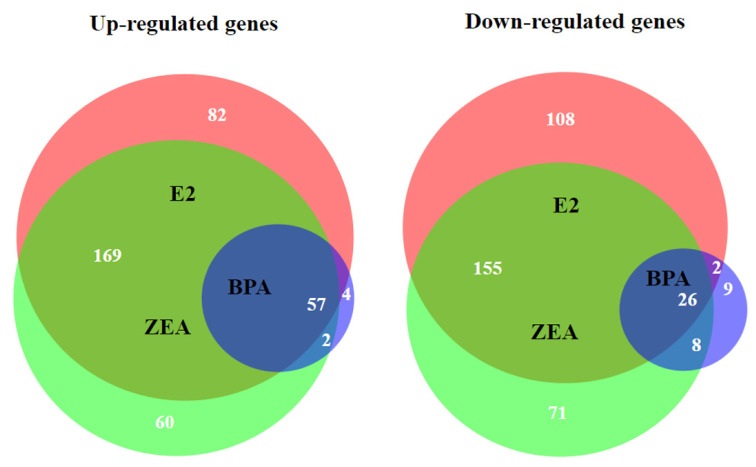
Venn diagrams representing the overlaps of up- (Log_2_FC > 1) and downregulated (Log_2_FC < −1) genes in response E2 (red), ZEA (green) and BPA (purple) treatment.

Those genes were selected for further analysis, whose expression showed at least two-fold up- or downregulation (Log_2_FC > 1 or Log_2_FC < −1) in response to estrogen exposure. In order to characterize the overlapping transcriptomic changes caused by E2, ZEA and BPA exposure, Venn diagrams were created. A total of 83 DEGs showed altered expression in response to all the estrogens tested; among them, 57 were up- and 26 were downregulated (Figure 3, Table 1). It is important to mention that several of these DEGs were also found to be involved in the estrogen response of other cell lines, as was confirmed in previous studies (Table 1).

The effect of E2 and ZEA proved to be highly comparable: the expression of 407 DEGs changed significantly in response to both E2 and ZEA treatment (Figure 3; Supplementary Materials Table S3). We can also conclude that the overlap in gene expression was more evident in the case of the upregulated genes than in the case of the downregulated genes (Figure 3). Most of the BPA-responsive DEGs also showed response to E2 and/or ZEA. A change in the expression of 13 genes was specific for BPA exposure only (Figure 3; Supplementary Materials Table S3).

**Figure 4:**
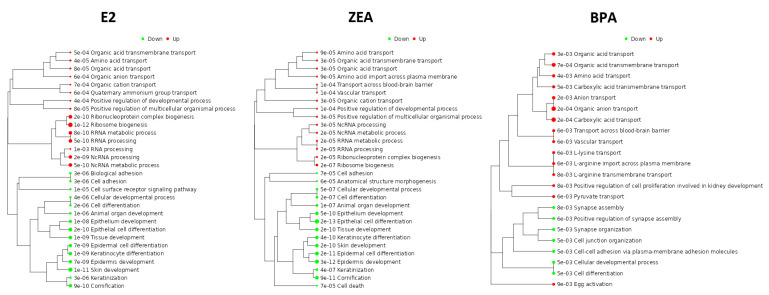
The functional annotation of up- (Log_2_FC > 1) and downregulated (Log_2_FC < −1) genes in response to E2, ZEA or BPA treatment. Functional enrichment analysis was made by iDEP.96 using the GO_BP database.

To assess the consequences of the estrogen-induced changes in gene expression, functional enrichment analysis using the GO_BP and Reactome databases was carried out in the case of the up- and downregulated DEGs in order to identify the induced and repressed pathways in response to estrogen treatments. According to the GO_BP database, several pathways related to amino acid, organic acid or ion transport, showed induction in response to all the treatments (Figure 4). It is also important to mention that enriched pathways involved in RNA processing (NcRNA processing, RRNA processing, NcRNA metabolic process) were also found. On the contrary, several pathways involved in the maintenance of epithelial phenotype (epithelial cell differentiation, epidermis development) and cell adhesion (biological adhesion, cell adhesion) were downregulated (Figure 4). These results were in good agreement with the analysis that was based on the Reactome database. During this analysis, only the top 20 hits were considered according to their significance (*p*-values; Supplementary Materials Table S4). The enriched pathways were categorized to cellular processes. Our analysis revealed high functional overlap between the pathways altered by E2 and ZEA treatment. Obviously, this might be the consequence of the significant overlap between the up- and downregulated DEGs in response to these molecules (Figure 3; Supplementary Materials Table S4). The enrichment of the upregulated genes proved to be the highest in pathways related to cell cycle and RNA metabolism (e.g., mitotic G1 phase and G1/S transition, rRNA processing in the nucleus and cytosol; Supplementary Materials Table S4). Furthermore, pathways involved in amino acid transport (amino acid transport across the plasma membrane) and estrogen-dependent gene expression also showed considerable enrichment (Supplementary Materials Table S4). The DEGs upregulated by BPA were also enriched in processes related to cell cycle, amino acid metabolism and estrogen-dependent gene expression. However, no significant enrichment was observed in RNA metabolism (Supplementary Materials Table S4). Similar phenomenon was found in the case of the downregulated genes, whose enrichment correlated better in the E2 and ZEA comparison. Top pathways that showed enrichment of the downregulated DEGs were involved in keratinization and cell junction organization. Genes involved in the regulation of programmed cell death also showed a significant enrichment (Supplementary Materials Table S4). Note that the DEGs downregulated by BPA were significantly enriched in fewer processes according to their *p*-value, so in this analysis only 11 pathways are presented (Supplementary Materials Table S4).

### 2.3. E2, ZEA and BPA Altered the Expression of miRNAs

**Figure 5:**
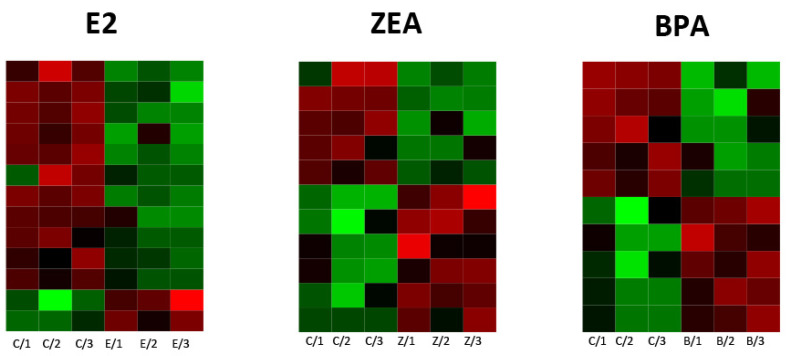
A heatmap of up- (Log_2_FC > 1) and downregulated (Log_2_FC < −1) miRNAs in response to E2, ZEA and BPA treatments. The expression profile of the three replicas is presented (C: nontreated control; E: E2-treated; Z: ZEA-treated; B: BPA-treated).

In our previous studies, estrogens were able to affect the expression of several miRNAs [23,24,25]. In order to identify more miRNAs that might be involved in the estrogen response we performed miRNA sequencing. The same experimental conditions were used as was the case with mRNA sequencing. The expression of 74, 47 and 73 miRNAs changed significantly in response to E2, ZEA or BPA treatment, respectively (*p* < 0.05, Supplementary Materials Table S5). Fold changes were characterized by Log_2_FC values that were calculated by the iDEP.96 web tool, which uses the DESeq2 algorithm. We considered miRNAs that showed at least two-fold up- or downregulation, as previously seen in the case of mRNA sequencing data. According to the log_2_FC values, 13, 11 and 10 miRNAs showed at least two-fold up- or downregulation in their expression (Figure 5; Table 2). Among the identified miRNAs the expression of miR-6795-3p, miR-5008-5p, miR-501-5p and miR-197-5p was downregulated in response to at least two treatments (Table 2).

**Figure 6:**
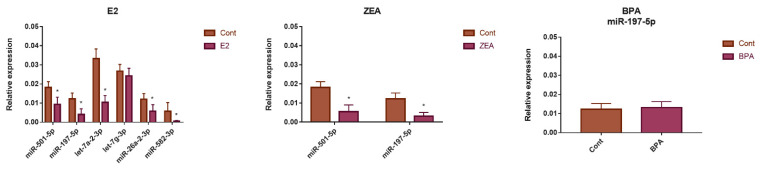
A study of the changes of miRNA expression in response to E2, ZEA and BPA exposure by qPCR. The relative expression values of selected miRNAs are presented in the nontreated control (Cont), and treated (E2, ZEA or BPA) samples. * *p* < 0.05, Student *t*-test.

We applied the qPCR method in order to strengthen the expression pattern of some miRNAs. For this purpose, six miRNAs (miR-501-5p, let-7a-2-3p, let-7g-3p, miR-26a-2-3p, miR-197-5p and miR-582-3p) were chosen, whose role in cancer progression was previously experimentally confirmed in other cell lines [67,68,69,70,71]. According to our qPCR results, the downregulation of miR-501-5p, let-7a-2-3p, miR-26a-2-3p and miR-197-5p was confirmed in response to E2 exposure (Figure 6). It is important to mention that the downregulation of miR-582-3p was seen by qPCR in contrast to its upregulation in the sequencing data (Table 2; Figure 6). The downregulation of miR-501-5p and miR-197-5p was also observed in response to ZEA exposure by both qPCR and miRNA sequencing (Figure 6; Table 2). However, the expression of miR-197-5p did not change significantly in response to BPA exposure in the qPCR experiments, in contrast to its downregulation, according to the miRNA sequencing data (Figure 6; Table 2).

### 2.4. Alterations in miRNA Expression Induced by E2, ZEA and BPA Might Support Tumor Growth and Migration

**Figure 7:**
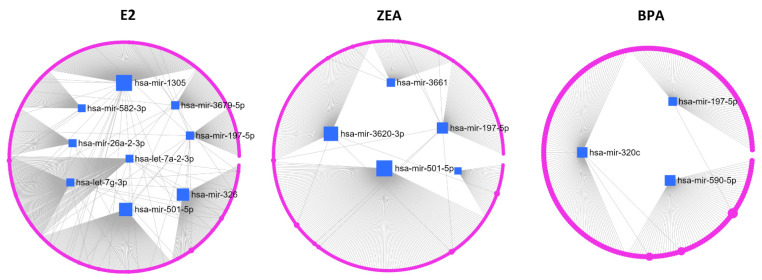
A network analysis of miRNAs showed up- (Log_2_FC > 1) and downregulation (Log_2_FC < −1) in response to E2, ZEA or BPA treatment. Blue squares represent the studied miRNAs, pink dots represent interacting proteins.

Due to the fact that a single miRNA targets several mRNAs, and a mRNA can be targeted by several miRNAs, biological processes involving miRNAs are very likely regulated by miRNA–protein interaction networks. Thus, we constructed a combined miRNA–protein network by the miRNet webtool using the miRTarBase v8.0 database containing experimentally validated miRNA–protein interactions. The largest network considering the number of miRNA target nodes was created by the E2-responsive differently expressed miRNAs (Figure 7; Table 3). The miRNAs that responded to ZEA and their targets were also provided a large network (Figure 7; Table 3). It is important to mention that miR-501-5p and miR-197-5p—whose downregulation was observed in response to E2 and ZEA by both qPCR and miRNA sequencing—were characterized by a high degree and betweenness centrality values confirming their biological importance in the created networks due to their high connectivity with the other miRNAs and target genes (Table 3). The network containing the smallest number of nodes was generated from the BPA-responsive miRNAs (Figure 7; Table 3). Note that some of our identified miRNAs were not present in the database, so the absence of these miRNAs in the networks is explained by the lack of information.

**Figure 8:**
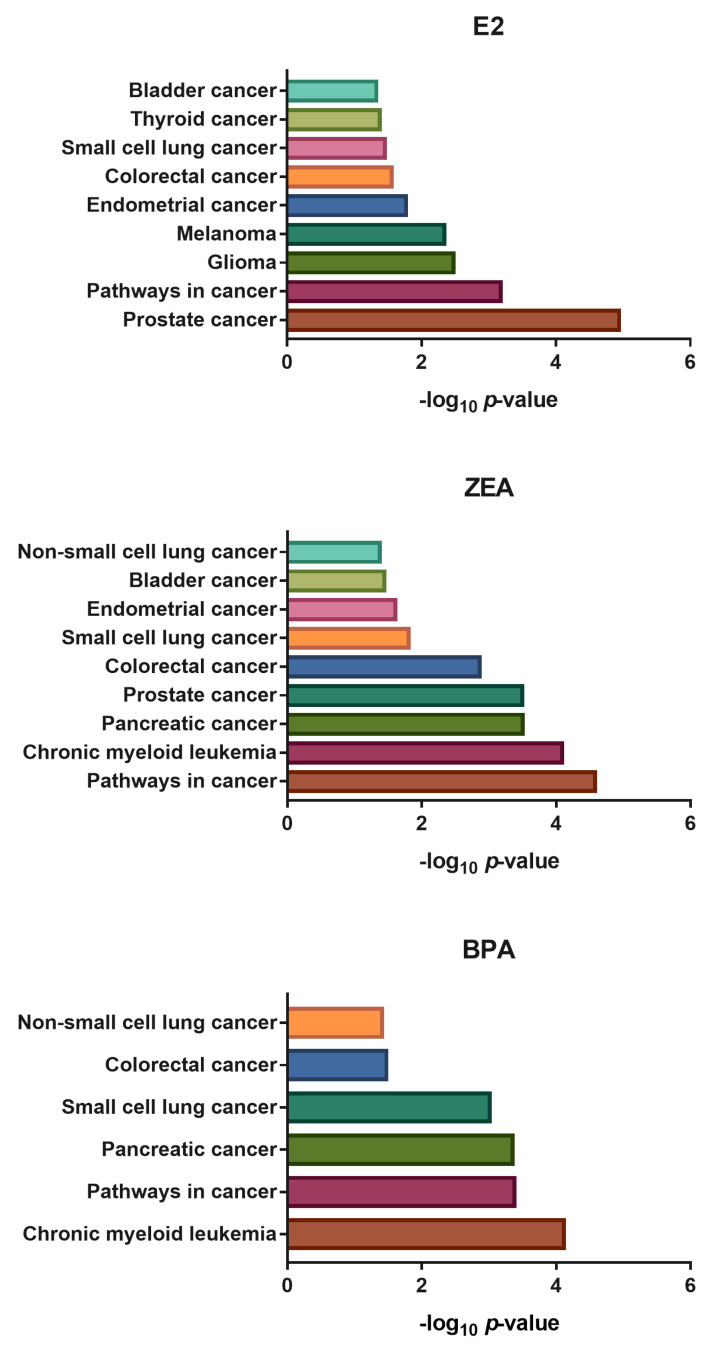
The functional annotation of miRNA–protein interacting networks created by miRNAs showed up- (Log_2_FC > 1) and downregulation (Log_2_FC < −1) in response to E2, ZEA or BPA treatment. Functional enrichment analysis was made by miRNet using the KEGG database.

Functional enrichment analysis was also created based on miRNA–interacting–proteins using the GO_BP, KEGG and Reactome databases. According to the GO_BP database, the differently expressed miRNAs might function in the regulation of several processes related to tumor growth and invasion (Supplementary Materials Tables S6–S8). Among others, the targets of miRNAs responded to E2 were enriched in the regulation of cell proliferation (*p* = 0.000649), cell cycle (*p* = 0.00107), cellular metabolic processes (*p* = 0.000339), apoptosis (*p* = 0.000525), cell adhesion (*p* = 0.000898) and cell migration (*p* = 0.00176) (Supplementary Materials Table S6). In the case of ZEA, the miRNA targets were enriched in the regulation of biosynthetic processes (*p* = 0.00229), cellular metabolic processes (*p* = 0.00258), G1 phase mitotic cell cycle (*p* = 0.00334), growth (*p* = 0.00883), programmed cell death (*p* = 0.000466), apoptosis (*p* = 0.000396) and cell migration (*p* = 0.012) (Supplementary Materials Table S7). In the case of BPA-responsive miRNAs, their targets were enriched in the regulation of biosynthetic processes (*p* = 0.00000122), developmental growth (*p* = 0.0000117), cellular metabolic processes (*p* = 0.0000558), cell differentiation (*p* = 0.00162), cell growth (*p* = 0.0018), epithelial to mesenchymal transition (*p* = 0.00617) and cell migration (*p* = 0.00592) (Supplementary Materials Table S8). Furthermore, a functional enrichment analysis of miRNA–interacting–proteins resulted in various pathways that are related to the progression of several cancer types, including hormone-related cancers, according to the KEGG database (e.g., prostate cancer, endometrial cancer, thyroid cancer) (Figure 8).

## 3. Discussion

During recent years, a growing interest has been invested into the understanding of the effect of xenoestrogens (including BPA and ZEA) in human health. Human exposure to these molecules is considered to be high as it was found that both BPA and ZEA are detectable in body fluids [16,72]. Furthermore, these molecules may also be toxic for the offspring, due to their presence in the amniotic fluid or breast milk [16,73]. In our previous studies, ZEA and BPA were able to exert an estrogen response in the PEO1 human ovarian cell line [23]. According to our new mRNA- and miRNA sequencing data, this effect is based on the significant transcriptomic changes induced by these compounds. To the best of our knowledge, ours is the first study to provide such data in a human ovarian cell line. Intensive changes in the expression of several genes in response to these molecules were also previously observed in other cell lines [74,75,76,77,78,79,80,81,82]. We can conclude that the effect of ZEA was comparable to the effect of physiological E2, with regard to the effective dose, and also the expression level of the differently expressed genes. It is in good agreement with the observation that ZEA was able to interact with ERα, and had a confirmed strong agonist activity in the ERE-luc reporter assays in several cell lines [83]. The application of 10 nM of E2 and ZEA also previously exerted a well-comparable induction of estrogen responsive genes in MCF-7 cells [76]; the carcinogenic effect of ZEA was also suggested by others [15]. In contrast to the strong estrogenic activity of ZEA, the effect of BPA proved to be less significant. This is in good agreement with the results of others [81,84] and can be explained by the observation that BPA has a lower affinity to ERs than E2, thus BPA is considered to be a weak estrogen [83,85].

**Figure 9:**
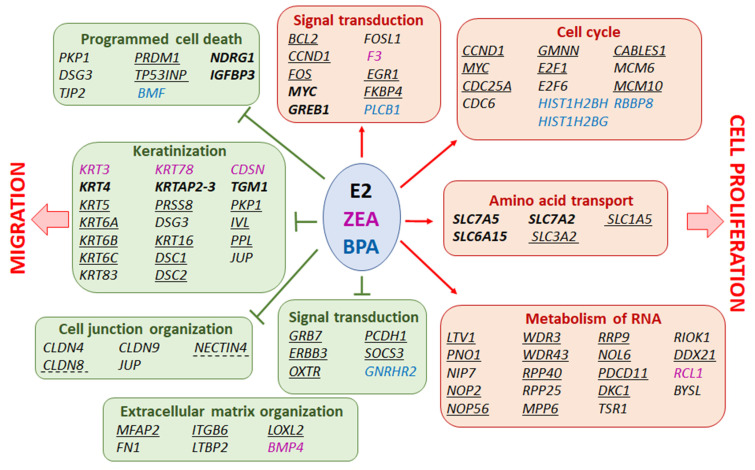
A summary of altered pathways and interacting genes in response to E2, ZEA and BPA exposure. Genes in black color responded to E2 treatment. Bold genes responded to all treatments. Underlined genes responded to both E2 and ZEA. Genes underlined by a dashed line responded to E2 and BPA. Genes in pink color responded to ZEA treatment. Genes in blue color responded to BPA treatment. The construction of the figure was based on our functional enrichment analysis performed by the Reactome database (Supplementary Materials Table S4).

According to our functional enrichment analysis of DEGs, we found E2, ZEA and BPA induced the expression of several genes that are involved in the regulation of cell proliferation, which is in good agreement with our previous studies [23]. It is important to mention that both ZEA and BPA were previously able to induce cell proliferation in ovarian, colon, prostate and breast cancer cell lines [14,74,75,86,87,88,89,90]. Furthermore, the exposure to these molecules induced several genes involved in RNA metabolism, especially in the processing of rRNAs, which suggests an increased rate of ribosome biogenesis during estrogen exposure. This might support cell proliferation by increasing the rate of protein synthesis that has a prominent role in driving tumorigenesis in cancer cells [91]. Estrogen exposure also induced ribosome biogenesis in breast cancer cells, as was shown previously [92]. The increased rate of amino acid transport might also be important in providing the monomers for protein synthesis, as well as providing energy for rapidly growing cells. It is important to mention that the inhibition of both ribosome biogenesis or amino acid transport are considered to be promising therapeutic strategies in cancer [92,93]. On the contrary, pathways involved in keratinization, extracellular matrix organization or tight-junction formation showed downregulation in response to estrogen exposure. Keratins are considered to be important intermedier filaments of epithelial cells that affect their integrity, trafficking, apical-basal polarization or motility, and are frequently used diagnostic markers in epithelial cancer, including ovarian cancer [94]. Furthermore, the downregulation of these molecules might support epithelial–mesenchymal transition, which is an essential process for the migration of epithelial tumors [95]. The reorganization of the extracellular matrix and the loss of tight junctions—processes which were shown to be downregulated in our study—are also considered to be important in the above-mentioned process [96]. This hypothesis is also supported by our previous phenotypic studies, where E2, ZEA and BPA induced cell migration by downregulating E-cadherin [23]. The affected processes, as well as the contributing genes, are summarized in Figure 9.

Due to the weaker estrogen-like activity of BPA, this molecule might be suitable for the identification of highly responsive genes in the estrogen action of ovarian cells. We could identify 83 genes that were significantly up- or downregulated in response to all the treatments, and might have a key role in the estrogen response of ovarian cells. It is also supported by the fact that the role of many of these DEGs in estrogen action was also suggested by others, in several other cell lines (Table 1). It is also important to mention that the expression of *GREB1*, *CA12*, *DHRS2*, *RBBP8*, *SLC7A5*, *PLAT*, *AFF3* and *PDGFRL* genes were associated with ERα positivity and/or the response to endocrine therapy in breast cancer [30,37,41,43,46,48,97,98]. This raises their possible application in the diagnostics of ERα-positive cancers. We could also identify 130 and 13 genes that showed altered expression in response to ZEA or BPA only, respectively. These might be useful candidates in the verification of ZEA and/or BPA exposure. Among these genes the differential expression of *CDKN1C* [99], *HMOX1* [100], *BMP4* [101], *GJB2* [102], *BDNF* [77] and *RGS16* [101] following exposure to ZEA was also previously reported. In those genes that responded to BPA only the expression change of *NNAT* was previously observed [103]; however, further studies are required in order to validate the diagnostic efficiency of these genes as biomarkers.

MicroRNAs have become the focus of interest in cancer research for the following reasons: (i) miRNAs might function as oncogenes or tumor suppressors, and thus might be involved in the development of cancer; (ii) the miRNA expression of normal cells differs from the miRNA expression of cancer cells, which makes them promising biomarker candidates; and (iii) modulating the miRNA content of tumor cells by replenishing tumor-suppressor miRNAs, or by the inhibition of oncogenic miRNAs, is considered to be a promising future therapeutic strategy in cancer [104,105,106]. We aimed to identify miRNAs that might assist the proliferative action of estrogens in human ovarian cells. We could identify several miRNAs that showed altered expression to the treatments and might interact in the enhancement of cell proliferation and migration, according to our functional enrichment analysis. The effect of E2, ZEA or BPA to miRNA expression was also confirmed by others [107,108,109,110,111,112,113]. It is important to note that although the largest miRNA target gene interaction network was created between the miRNAs that responded to E2, in our analysis this might be a consequence of the lack of knowledge about several miRNAs which responded to ZEA or BPA. For this reason, we could only make careful conclusions from the comparison of the effect of these molecules on miRNA expression, and about the biological consequences of the induced changes in the expression of their target genes.

During the qPCR experiment we tested the expression of six miRNAs. We could strengthen the downregulation of let-7a-2-3p, miR-501-5p, miR-26a-2-3p and miR-197-5p in response to E2 treatment, and the downregulation of miR-197-5p and miR-501-5p in response to ZEA by qPCR. Furthermore, according to our qPCR results, miR-582-3p was downregulated in response to E2, in contrast to its upregulation in the miRNA sequencing experiments. The fact that we could not strengthen the expression change of all the tested miRNAs should be addressed as a limitation of our study. Because qPCR is considered to be the most reliable method for the quantification of miRNA expression, our discussion about individual miRNAs was based on the results obtained by this method. These results are in good agreement with the observations of others. The role of let-7a-2-3p, miR-501-5p, miR-26a-2-3p and miR-197-5p in cancer progression was previously confirmed in other cell lines [67,68,69,70], and the repression of let-7a and miR-26a in response to E2 treatment was also observed in breast cancer [107]. Furthermore, the inhibition of miR-582-3p by E2 was also confirmed in breast cancer cells [107] and this miRNA suppressed the proliferation of ovarian cells, as was previously published [71]. We hypothesize that the downregulation of these miRNAs might support the proliferative action of estrogens.

## 4. Conclusions

Human and animal exposure to xenoestrogens, such as BPA or ZEA, will tend to grow in the future. The enormous amount of plastic pollution in the environment is considered to be a relevant risk factor for the contamination of ground water by molecules derived from plastics, and for the production of microparticles that might contaminate humans/animals through their inhalation. Furthermore, the mycotoxin contamination of food and feed products shows an increasing trend due to the increasing incidence of fungal infections in agriculture [15,17]. For this reason, understanding their effect on human health has particular importance. We conclude that both ZEA and BPA exert transcriptomic changes including cell proliferation, ribosome biogenesis or epithelial mesenchymal transition that favors cancer progression and metastasis formation, among which the effect of ZEA proved to be more relevant. The limitation of our study is that it is based on a cancer cell line that carries some differences in physiological parameters from healthy cells. However, it is important to state that increasing the proliferative potential of cells that have already undergone cancer transformation, and supporting their metastasis formation ability, might also be relevant in cancer progression. We would like to draw attention to the fact that, so far, ZEA is categorized as a group 3 compound: “Not classifiable as to its carcinogenicity to humans”, according to IARC guidelines [114]. Our data suggest that human exposure to ZEA requires more attention in the future, due to its high estrogenic activity and its possible role in cancer progression.

## 5. Materials and Methods

### 5.1. Cell Culturing

The PEO1 human epithelial ovarian cell line that was used in this study was purchased from Merck (Darmstadt, Germany; ECACC, Salisbury, UK). The expression of ERα and its estrogen sensitivity was confirmed in our previous studies [23,24,25]. The PEO1 was cultured in RPMI1640 medium supplemented with 10% fetal bovine serum (FBS), 1% L-glutamine, 100 µg/mL streptomycin and 100 U/mL penicillin (Corning, New York, NY, USA); 37 °C, 90% humidity, 5% CO_2_. In the transcriptional studies, cells were harvested by trypsinization and plated in the above-mentioned medium. At 24 h after plating, the medium was replaced with PRF-RPMI1640 supplemented with 5% DCC-FBS and cells were incubated for another 24 h. This step was necessary in order to reduce the confounding effect of phenol red or estrogens present in the conventional medium. After the incubation period, cells were treated with E2, ZEA and BPA (Merck, Darmstadt, Germany) in 10 and 100 nM final concentrations (dissolved in DMSO). The estrogen supplementation was considered as 0 h for the gene expression studies.

### 5.2. MRNA Isolation and Quantification by qPCR

In order to analyze the transcriptome in PEO1, 10^4^ cells were plated to 24-well plates and at 0 h time the cultures were supplemented with E2, ZEA or BPA, as described above. At 8 h after the treatment, total RNA was isolated from the cells using the Quick-RNA MiniPrep Kit (Zymo Research, Irvine, CA, USA), according to the instructions of the manufacturer. The reverse transcription of RNA to synthetize cDNA was performed using 500 ng total RNA as a template by the Maxima First Strand cDNA Synthesis Kit (Thermo Fisher Scientific, Walthman, MA, USA), according to the instructions of the manufacturer. A NanoDrop LITE Spectrophotometer (Thermo Fisher Scientific, Walthman, MA, USA) was used for the quantification of total RNA or cDNA concentrations. The expression of *GREB1*, *CA12*, *DEPTOR*, *AGT*, *RBBP8NL*, *MYC*, *EGR1*, *NOLC1*, *RRP12*, *BLNK*, *TGMI*, *KRT4*, *BMF*, *CD24*, *NOTCH3*, *GBP3* and *MYBL1* was determined by qPCR using the Maxima™ SYBR Green qPCR Master Mix (Thermo Fisher Scientific, Walthman, MA, USA), in a Lightcycler 96 instrument (Roche, Pleasanton, CA, USA), following the instructions of the manufacturer. Primer sequences are presented in Supplementary Materials Table S9. The mRNA expression values were normalized to *GAPDH* expression and the results of four independent experiments were used to calculate gene expression, which proved to be a reliable method in our previous studies [23,25]. Changes in the relative expression level of the target genes in response to estrogen treatment was determined by the 2^−∆∆Ct^ formula, where ∆∆Ct = ∆Ct treated sample − ∆Ct control sample. Fold changes (FC) were presented in a log_2_ scale. Figures and statistics were made by the GraphPad Prism 7.0.

### 5.3. MiRNA Isolation and Quantification by qPCR

In order to determine miRNA expression, 10^4^ PEO1 cells were plated to 24-well plates and treated with E2, ZEA or BPA, as previously described. Further, 8 h after the treatment, total RNA, including small RNAs, was isolated using the miRNeasy Kit (Qiagen, Hilden, Germany), following the instructions of the manufacturer. Total RNA concentration was quantified by the NanoDrop LITE Spectrophotometer (Thermo Fisher Scientific, Walthman, MA, USA). For the determination of miRNA expression, the miRCURY LNA workflow was applied (Qiagen, Hilden, Germany). The reverse transcription of 20 ng RNA was performed by the miRCURY LNA RT kit (Qiagen, Hilden, Germany), following the instructions provided by the manufacturer. The expression of miR-501-5p, let-7a-2-3p, let-7g-3p, miR-26a-2-3p, miR197-5p and miR-582-3p was quantified using miRCURY LNA miRNA PCR assays with a miRCURY LNA SYBR Green PCR Kit (Qiagen, Hilden, Germany), in a Lightcycler 96 instrument (Roche, Pleasanton, CA, USA), following the instructions provided by the manufacturer. The expression values of miRNAs were normalized to miR-103-3p expression, which proved to be a reliable internal control in our previous studies [23,24,25,115]. The relative expression values of miRNAs were calculated from the results of four independent experiments applying the 2^−∆Ct^ formula. Figures and statistics were made by the GraphPad Prism 7.0.

### 5.4. Transcriptomic Analysis by RNA Sequencing

Library preparations, sequencing and primary data analysis were performed by the Genomic Medicine and Bioinformatics Core Facility (Department of Biochemistry and Molecular Biology, Faculty of Medicine, University of Debrecen), using the Illumina NextSeq500 platform. Three replicates were used for sequencing: three nontreated control and three treated (with E2, ZEA or BPA) samples. The same culturing conditions and isolation protocols were applied as in the qPCR studies, in order to obtain comparable results. Namely, the miRNeasy Kit (Qiagen, Hilden, Germany)—which is optimized for studies about miRNAs—was used for RNA isolation in the miRNA sequence experiment, and the Quick-RNA MiniPrep Kit (Zymo Research, Irvine, CA, USA)—which is suitable for studies based on mRNAs—was used in the case of mRNA sequencing, as in our previous studies [23,24,25], according to the protocols provided by the manufacturers. The quality of RNA samples was determined by the Agilent Bio Analyzer with the Eukaryotic Total RNA Nano Kit, following the instructions of the manufacturer. Only those samples were used for library preparation, in which the RNA integrity number (RIN) value proved to be higher than seven. Library preparation for mRNA sequencing was performed with the NEBNext Ultra II RNA Sample Prep kit (New England BioLabs, Ipswich, MA, USA), according to the instructions of the manufacturer. Sequencing libraries for small RNA sequencing were generated using the NEBNext Multiplex Small RNA Perp Set for Illumina (1-48) 96 rxn kit (New England BioLabs, Ipswich, MA, USA), according to the instructions provided by the manufacturer. Sequencing was performed on the Illumina NextSeq 500 instrument (Illumina, San Diego, CA, USA), using single-end 75 cycles sequencing (for mRNA sequencing) or 50 bp sequencing run (for small RNA sequencing). During sequencing, 16–23 million reads were generated. In the case of sequencing data, the quality score proved to be Q30 > 90% in all samples. During RNA sequencing data analysis, the GRCh38.p13 (HG38: GCF_000001405.39_GRCh38.p13_genomic.fna) human reference genome was used in the alignment of raw sequencing data using the algorithm HISAT2 [116,117], and BAM files were generated in the case of mRNA sequencing. During miRNA sequencing, the Novoalign algorithm was used for alignment to the reference genome. The StrandNGS software (www.strand-ngs.com) was applied for downstream analysis that included normalization by the DESeq algorithm using the BAM files generated previously, and the generation of FPKM values. Moderated *t*-test (with Benjamini-Hochberg FDR correction) was used in order to identify expression changes in response to estrogen exposure relative to the nontreated control samples (*p* < 0.05).

During bioinformatic analysis, the generated FPKM values were applied in order to calculate log_2_FC values by the iDEP.96 web tool (http://bioinformatics.sdstate.edu/idep96; accessed on 28 November 2022). Note that the cut-off was set to 1 or higher in at least one sample, in order to exclude genes or miRNAs with low expression. The distribution of FPKM values in the case of the mRNA and miRNA samples were presented in Supplementary Materials Figure S2 and S3, respectively. Bioinformatic analysis included the calculation of log_2_FC values, the generation of heatmaps and MA plots. Pathway enrichment analysis for the up- and downregulated genes was also performed by the iDEP.96 webtool (using the GO_BP) and the Reactome databases (https://reactome.org/PathwayBrowser/; accessed on 16 January 2023) in gene expression analysis. Venn diagrams were created by BioVenn (https://www.biovenn.nl/index.php; accessed on 5 December 2022). In the case of the miRNAs, log_2_FC values were also calculated by the iDEP.96 webtool. To carry out network construction and pathway enrichment analysis with the up- and downregulated miRNAs and their target genes, the miRNet software (https://www.mirnet.ca; accessed on 12 December 2022) was applied using the GO_BP, KEGG and Reactome databases.

## Figures and Tables

**Table 1:** A list of genes and Log_2_FC values (with FDR *p*-value) that showed up- (Log_2_FC > 1) and downregulation (Log_2_FC < −1) in response to E2, ZEA and BPA. References for their hypothetic or validated role in estrogen action are also presented.

Gene	E2		ZEA		BPA		Reference
	Log_2_FC	FDR	Log_2_FC	FDR	Log_2_FC	FDR	
*GREB1*	4.71	7.39 × 10^−11^	4.79	3.95 × 10^−8^	3.64	3.61 × 10^−12^	[26,27]
*KCNF1*	4.19	2.15 × 10^−10^	4.23	7.10 × 10^−8^	3.27	8.96 × 10^−12^	
*DEPTOR*	4.17	7.39 × 10^−11^	3.77	5.25 × 10^−8^	2.79	8.96 × 10^−12^	[28]
*CA12*	4.08	7.39 × 10^−11^	4.10	2.98 × 10^−8^	2.74	4.80 × 10^−12^	[29,30]
*AGT*	3.31	2.69 × 10^−10^	3.03	5.25 × 10^−8^	2.21	1.38 × 10^−11^	[31]
*MGAT3*	3.31	2.15 × 10^−10^	3.25	3.15 × 10^−8^	2.03	4.05 × 10^−12^	
*CBLN1*	3.29	2.71 × 10^−10^	3.10	1.20 × 10^−7^	2.64	3.74 × 10^−12^	[32]
*OLFM1*	3.28	1.43 × 10^−9^	3.14	6.37 × 10^−7^	1.79	1.29 × 10^−8^	[33,34]
*ARHGAP26*	3.25	2.22 × 10^−10^	3.35	3.95 × 10^−8^	2.33	8.95 × 10^−12^	
*SEZ6*	3.21	1.04 × 10^−8^	3.18	1.61 × 10^−7^	2.49	3.15 × 10^−12^	
*PPP1R1A*	3.13	2.08 × 10^−10^	2.82	2.98 × 10^−8^	1.26	4.58 × 10^−10^	
*CADM1*	3.11	2.69 × 10^−10^	3.13	3.37 × 10^−8^	2.11	6.99 × 10^−12^	[35]
*PIPOX*	3.05	9.83 × 10^−8^	2.95	5.97 × 10^−6^	2.36	4.42 × 10^−8^	
*DHRS2*	2.98	5.99 × 10^−10^	2.78	1.17 × 10^−7^	1.50	2.70 × 10^−9^	[36,37]
*PKDCC*	2.97	6.56 × 10^−10^	2.95	2.98 × 10^−8^	2.60	4.05 × 10^−12^	
*SLC7A2*	2.93	1.43 × 10^−9^	2.92	2.89 × 10^−7^	2.04	2.70 × 10^−10^	[38,39,40]
*HPDL*	2.81	2.51 × 10^−8^	2.63	3.42 × 10^−6^	1.82	7.26 × 10^−8^	
*CABLES1*	2.73	2.94 × 10^−10^	2.55	3.14 × 10^−8^	1.58	2.63 × 10^−11^	
*RHOBTB1*	2.72	9.85 × 10^−10^	2.61	8.73 × 10^−8^	1.86	9.33 × 10^−11^	
*RBBP8*	2.71	2.69 × 10^−10^	2.60	3.95 × 10^−8^	1.92	1.49 × 10^−11^	[41]
*RUBCNL*	2.69	1.05 × 10^−8^	2.58	1.00 × 10^−6^	1.98	1.35 × 10^−8^	
*DIRAS3*	2.58	6.56 × 10^−10^	2.54	6.41 × 10^−8^	1.86	1.94 × 10^−11^	[42]
*LONRF2*	2.49	1.43 × 10^−9^	2.63	1.61 × 10^−7^	1.79	1.78 × 10^−10^	
*RIMS4*	2.35	3.09 × 10^−9^	2.11	9.53 × 10^−8^	1.32	3.73 × 10^−10^	
*SLC7A5*	2.32	9.02 × 10^−10^	2.13	8.63 × 10^−8^	1.29	1.20 × 10^−9^	[43,44]
*PLAT*	2.30	6.56 × 10^−10^	1.94	2.36 × 10^−6^	1.17	9.17 × 10^−9^	[45]
*CISH*	2.18	1.06 × 10^−8^	2.13	6.54 × 10^−7^	1.77	2.70 × 10^−10^	
*AFF3*	2.11	1.77 × 10^−8^	2.38	6.58 × 10^−7^	2.04	2.11 × 10^−10^	[46,47]
*PDGFRL*	2.06	1.41 × 10^−8^	1.65	7.60 × 10^−7^	1.19	4.13 × 10^−9^	[48]
*RASGRP1*	2.03	2.41 × 10^−8^	2.11	8.43 × 10^−7^	1.65	9.16 × 10^−10^	
*BEND3*	2.00	1.47 × 10^−8^	1.99	9.05 × 10^−7^	1.34	7.40 × 10^−8^	
*PPARGC1B*	1.95	1.49 × 10^−7^	2.09	6.75 × 10^−6^	1.36	9.28 × 10^−8^	[49]
*MTHFD2P7*	1.93	3.40 × 10^−8^	1.61	8.43 × 10^−7^	1.36	1.40 × 10^−8^	
*ERICH3*	1.88	1.37 ×10^−7^	1.90	3.96 × 10^−6^	1.34	5.68 × 10^−8^	
*PLCB1*	1.87	9.19 × 10^−8^	1.79	2.36 × 10^−6^	1.01	5.31 × 10^−7^	[50]
*CDCA7*	1.85	1.04 × 10^−8^	1.74	4.73 × 10^−7^	1.32	2.00 × 10^−9^	[51,52]
*OLFML3*	1.84	1.41 × 10^−8^	1.79	6.54 × 10^−7^	1.08	1.20 × 10^−8^	
*SLC22A3*	1.84	9.64 × 10^−9^	1.81	2.89 × 10^−7^	1.47	1.31 × 10^−10^	
*EGR1*	1.78	2.67 × 10^−8^	1.71	9.58 × 10^−7^	1.22	9.03 × 10^−9^	[53,54]
*SLITRK4*	1.76	5.89 × 10^−8^	1.76	1.40 × 10^−6^	1.15	3.05 × 10^−7^	
*PNPT1P1*	1.73	8.04 × 10^−8^	1.19	3.83 × 10^−5^	1.46	4.42 × 10^−8^	
*RRS1*	1.72	1.49 × 10^−8^	1.49	9.13 × 10^−7^	1.12	2.30 × 10^−8^	[55]
*ZNF239*	1.70	4.87 × 10^−8^	1.55	2.42 × 10^−6^	1.14	3.21 × 10^−8^	
*NPY1R*	1.69	1.99 × 10^−8^	1.54	3.45 × 10^−6^	1.32	2.19 × 10^−9^	[36]
*SLC6A15*	1.66	1.75 × 10^−7^	1.72	2.65 × 10^−6^	1.27	3.28 × 10^−8^	
*TSEN2*	1.66	2.68 × 10^−7^	1.49	1.14 × 10^−5^	1.10	3.82 × 10^−7^	
*ZNF485*	1.64	5.17 × 10^−7^	1.77	9.08 × 10^−7^	1.38	3.22 × 10^−9^	
*MYC*	1.63	1.49 ×10^−8^	1.65	4.36 × 10^−7^	1.26	4.51 × 10^−10^	[56,57]
*NCR3LG1*	1.62	1.94 × 10^−7^−	1.86	3.42 × 10^−6^	1.34	8.40 × 10^−8^	[40]
*TFAP4*	1.61	1.36 × 10^−7^	1.58	2.23 × 10^−6^	1.10	5.87 × 10^−8^	
*KBTBD8*	1.59	2.61 × 10^−8^	1.55	9.05 × 10^−7^	1.01	1.29 × 10^−8^	
*SLC19A2*	1.57	8.04 × 10^−8^	1.58	9.05 × 10^−7^	1.01	1.51 × 10^−8^	
*LYAR*	1.56	5.51 × 10^−8^	1.33	1.40 × 10^−6^	1.02	5.39 × 10^−8^	
*KAZN*	1.46	4.96 × 10^−8^	1.45	9.08 × 10^−7^	1.09	9.52 × 10^−9^	
*PUS7*	1.41	5.51 × 10^−8^	1.35	9.28 × 10^−7^	1.03	9.66 × 10^−9^	[58]
*ERG*	1.40	1.84 × 10^−8^	1.20	4.52 × 10^−5^	1.03	7.90 × 10^−8^	
*NXNL2*	1.28	1.69 × 10^−6^	1.25	4.68 × 10^−5^	1.27	8.75 × 10^−8^	
*CLDN8*	−1.32	3.21 × 10^−7^	−2.11	5.65 × 10^−7^	−1.03	3.19 × 10^−7^	[59,60]
*LINC01133*	−1.42	2.42 × 10^−7^	−1.26	5.64 × 10^−6^	−1.01	2.91 × 10^−7^	
*MFAP2*	−1.55	1.31 × 10^−7^	−1.80	5.65 × 10^−7^	−1.22	7.07 × 10^−8^	[61,62]
*IGSF9*	−1.60	5.90 × 10^−8^	−1.57	7.60 × 10^−7^	−1.03	4.15 × 10^−8^	
*KRTAP2-3*	−1.60	5.51 × 10^−8^	−1.35	9.58 × 10^−7^	−1.02	2.80 × 10^−8^	
*UNC5B-AS1*	−1.62	6.74 × 10^−8^	−1.38	5.76 × 10^−8^	−1.12	5.76 ×10^−8^	
*BMF*	−1.64	1.99 × 10^−8^	−1.65	4.90 × 10^−9^	−1.12	4.90 × 10^−9^	
*EPHB3*	−1.64	2.84 × 10^−8^	−1.62	4.08 × 10^−8^	−1.04	4.08 × 10^−8^	
*KRT4*	−1.71	2.13 × 10^−8^	−1.67	1.10 × 10^−8^	−1.01	1.10 × 10^−8^	[55]
*CYSRT1*	−1.72	1.69 × 10^−8^	−2.06	1.33 × 10^−8^	−1.10	1.33 × 10^−8^	
*RNF224*	−1.77	2.59 × 10^−8^	−1.96	1.68 × 10^−7^	−1.09	1.68 × 10^−7^	
*RASSF2*	−1.80	1.06 × 10^−6^	−1.64	4.32 × 10^−9^	−1.29	4.32 × 10^−9^	
*IKZF2*	−1.89	6.80 × 10^−8^	−1.47	3.52 × 10^−7^	−1.04	3.52 × 10^−7^	
*IGFBP3*	−1.89	1.04 × 10^−8^	−1.82	5.02 × 10^−9^	−1.06	5.02 × 10^−9^	[63]
*POU2F3*	−1.92	3.87 × 10^−7^	−1.99	1.46 × 10^−8^	−1.35	1.46 × 10^−8^	
*NDRG1*	−1.96	4.01 × 10^−9^	−1.97	2.70 × 10^−9^	−1.05	2.70 × 10^−9^	
*TGM1*	−2.00	2.22 × 10^−8^	−1.91	3.82 × 10^−8^	−1.00	3.82 × 10^−8^	[64]
*LINC01559*	−2.00	3.15 × 10^−7^	−1.90	1.34 × 10^−7^	−1.40	1.34 × 10^−7^	
*LRRC4*	−2.20	4.11 × 10^−8^	−2.21	2.76 × 10^−8^	−1.21	2.76 × 10^−8^	
*FILIP1L*	−2.33	1.49 × 10^−8^	−2.45	6.82 × 10^−10^	−1.22	6.82 × 10^−10^	
*C4ORF26*	−2.45	1.61 × 10^−7^	−2.18	2.30 × 10^−8^	−1.29	7.14 × 10^−8^	
*NECTIN4*	−2.78	1.43 × 10^−9^	−2.73	1.07 × 10^−10^	−1.70	1.07 × 10^−10^	
*AIM1L*	−2.95	1.04 × 10^−8^	−2.50	3.21 × 10^−10^	−1.48	3.21 × 10^−10^	
*BLNK*	−3.07	2.53 × 10^−7^	−3.03	2.70 × 10^−9^	−1.90	2.70 × 10^−9^	[65]
*RBBP8NL*	−3.19	1.28 × 10^−7^	−3.46	1.42 × 10^−8^	−2.07	1.42 × 10^−8^	[66]

**Table 2:** A list of up- (Log_2_FC > 1) and downregulated (Log_2_FC < −1) miRNAs (with FDR *p*-value) in response to E2, ZEA and BPA treatments.

E2			ZEA			BPA		
miRNA	Log_2_FC	FDR	miRNA	Log_2_FC	FDR	miRNA	Log_2_FC	FDR
let-7a-2-3p	−1.99	0.001	miR-6795-3p	−1.83	0.0002	miR-6795-3p	−1.66	0.029
miR-501-5p	−1.98	0.001	miR-3661	−1.68	0.052	miR-597-5p	−1.33	0.05
let-7g-3p	−1.98	0.017	miR-501-5p	−1.55	0.033	miR-197-5p	−1.21	0.046
miR-3679-5p	−1.95	0.001	miR-197-5p	−1.10	0.066	miR-5008-5p	−1.09	0.037
miR-26a-2-3p	−1.92	0.023	miR-5008-5p	−1.07	0.013	miR-320c	−1.03	0.065
miR-326	−1.65	0.035	miR-451a	1.09	0.039	miR-6879-3p	1.01	0.046
miR-6795-3p	−1.41	0.035	miR-3065-3p	1.33	0.095	miR-3934-5p	1.11	0.065
miR-1305	−1.27	0.092	miR-3620-3p	1.46	0.049	miR-590-5p	1.11	0.046
miR-197-5p	−1.27	0.026	miR-6806-3p	1.49	0.033	miR-636	1.22	0.05
miR-6765-3p	−1.16	0.035	miR-4747-3p	1.93	0.033	miR-6806-3p	1.45	0.046
miR-5008-5p	−1.05	0.026	miR-548u	2.34	0.013			
miR-582-3p	1.48	0.023						
miR-6775-3p	2.48	0.026						

**Table 3:** The degree and betweenness values of miRNA–protein interacting networks created by miRNAs showed up- (Log_2_FC > 1) and downregulation (Log_2_FC < −1) in response to E2, ZEA or BPA treatment. Note that the degree of a node means the number of connections it has to neighboring nodes. Betweenness centrality values indicating the number of shortest paths that go through the node of interest in the network.

E2			ZEA			BPA		
miRNA	Deg.	Betw.	miRNA	Deg.	Betw.	miRNA	Deg.	Betw.
miR-1305	195	141,840	miR-501-5p	158	48,544	miR-320c	108	19,875
miR-501-5p	158	107,695	miR-3620-3p	95	41,791	miR-197-5p	68	13,869
miR-326	138	96,436	miR-197-5p	68	27,959	miR-590-5p	66	20,688
let-7g-3p	106	43,469	miR-3661	49	16,350			
let-7a-2-3p	102	40,279	miR-451a	31	11,295			
miR-197-5p	68	46,136						
miR-3679-5p	63	45,319						
miR-26a-2-3p	59	42,825						
miR-582-3p	55	39,732						

## Data Availability

The data presented in this study are openly available in the Gene Expression Omnibus (GEO) database (https://www.ncbi.nlm.nih.gov/geo/) at the accession number: GSE224108.

## Supplementary Materials

The following supporting information can be downloaded at: https://www.mdpi.com/article/10.3390/toxins15020140/s1, Figure S1: Optimalization of E2, ZEA and BPA dose for transcriptomic studies by qPCR. Gene expression is presented as relative expression normalized to *GAPDH*. (* *p* < 0.05; ** *p* < 0.01; *** *p* < 0.001. One-way ANOVA, Dunnet-test); Figure S2: The distribution of FPKM values of differently expressed mRNAs in response to E2, ZEA and BPA according to the mRNA sequencing data (iDEP.96); Figure S3: The distribution of FPKM values of differently expressed miRNAs in response to E2, ZEA and BPA, according to the miRNA sequencing data (iDEP.96); Table S1: Differently expressed genes in response to E2, ZEA or BPA treatments; Table S2: Up- (log_2_FC > 1) and downregulated (log_2_FC < −1) genes in response to E2, ZEA and BPA treatments; Table S3: Differently expressed genes in response to E2, ZEA or BPA treatment only, as well as to E2 and ZEA, E2 and BPA, or ZEA and BPA treatments; Table S4: Functional gene enrichment analysis of genes up- or downregulated in response to E2, ZEA or BPA exposure. The top 20 pathway hits were selected based on their *p*-values. Note that in the case of genes downregulated to BPA, 11 pathways showed significant enrichment (*p* < 0.05). Processes highlighted with blue represent pathways that were enriched in response to two treatments. Processes highlighted with green represent pathways that were enriched in response to all three treatments; Table S5: Differently expressed miRNAs in response to E2, ZEA or BPA treatments; Table S6: Functional enrichment analysis of differently expressed miRNAs in response to E2. The target genes, as well as the results of the analysis, are presented according to the GO_BP, KEGG and Reactome databases; Table S7: Functional enrichment analysis of differently expressed miRNAs in response to ZEA. The target genes, as well as the results of the analysis, are presented according to the GO_BP, KEGG and Reactome databases; Table S8: Functional enrichment analysis of differently expressed miRNAs in response to BPA. The target genes, as well as the results of the analysis, are presented according to the GO_BP, KEGG and Reactome databases; Table S9: Primer sequences of 18 genes applied in the qPCR experiments.
