# Supplementary material for: Comparative Analysis of Transcriptomic Changes including mRNA and microRNA Expression Induced by the Xenoestrogens Zearalenone and Bisphenol A in Human Ovarian Cells
Source: Toxins (Basel). 2023 Feb 9;15(2):140. doi: 10.3390/toxins15020140 (PMC9967916; doi:10.3390/toxins15020140)
Supplement: Supplementary file 1 [file toxins-15-00140-s001.zip › Supplementary Figures.pdf]

toxins

Supplementary Materials: Comparative Analysis of Transcriptomic Changes including mRNA and microRNA Expression Induced by the Xenoestrogens Zearalenone and Bisphenol A in Human Ovarian Cells

Éva Márton, Alexandra Varga, András Penyige, Zsuzsanna Birkó, István Balogh, Bálint Nagy and Melinda Szilágyi

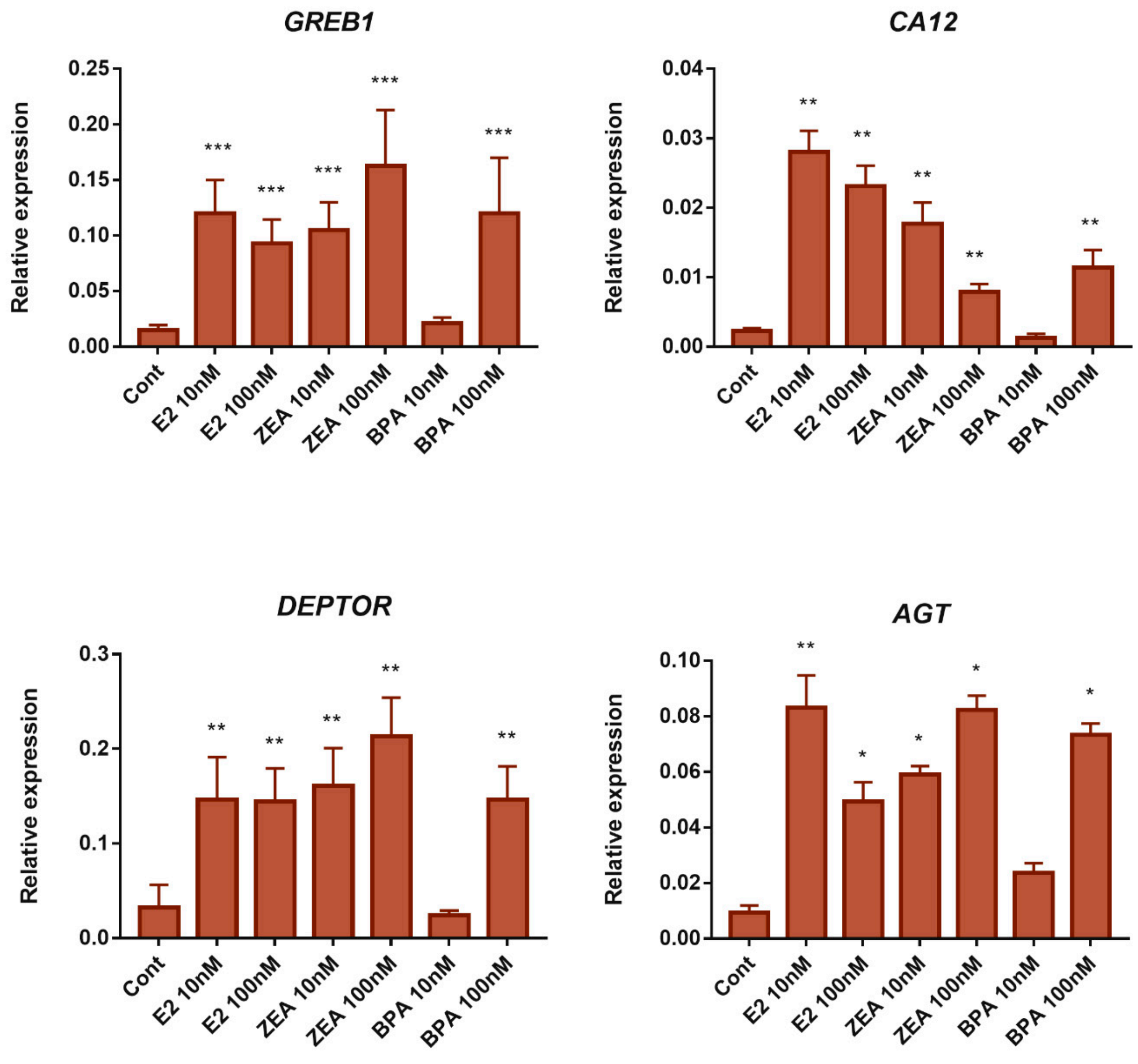

Supplementary Figure S1 Optimalization of E2, ZEA and BPA dose for the transcriptomic studies by qPCR. Gene expression is presented as relative expression normalized to *GAPDH*. *p<0.05; **p<0.01; ***p<0.001. One-way ANOVA, Dunnet-test.

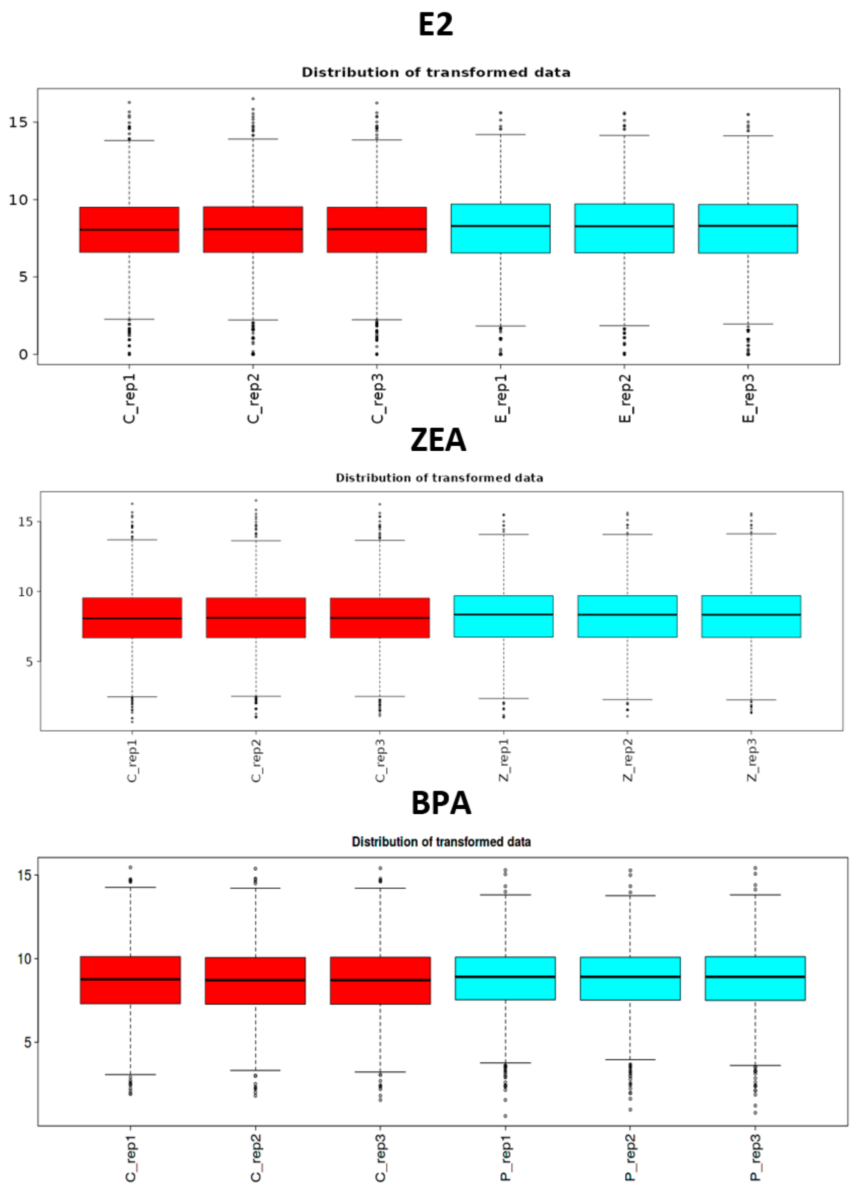

Supplementary Figure S2 The distribution of FPKM values of differently expressed mRNAs in response to E2, ZEA and BPA exposure according to the mRNA-seq data (iDEP.96).

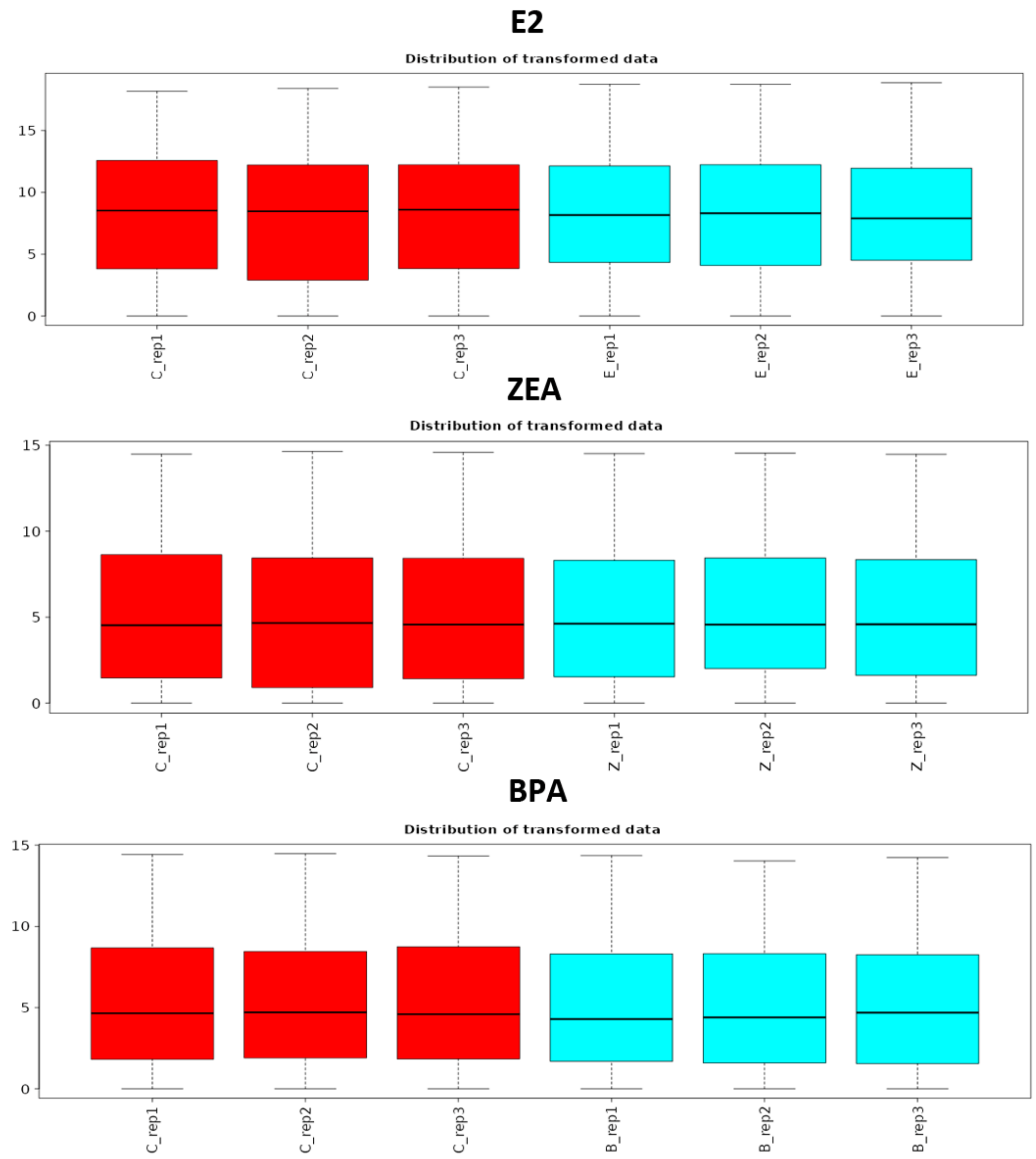

Supplementary Figure 3 The distribution of FPKM values of differently expressed miRNAs in response to E2, ZEA and BPA according to the miRNA-seq data (iDEP.96).
