# Supplementary material for: Comparative Analysis of Transcriptomic Changes including mRNA and microRNA Expression Induced by the Xenoestrogens Zearalenone and Bisphenol A in Human Ovarian Cells
Source: Toxins (Basel). 2023 Feb 9;15(2):140. doi: 10.3390/toxins15020140 (PMC9967916; doi:10.3390/toxins15020140)
Supplement: Supplementary file 1 [file toxins-15-00140-s001.zip › Supplementary Table S9.pdf]

toxins MDPI

Supplementary Materials: Comparative Analysis of Transcriptomic Changes including mRNA and microRNA Expression Induced by the Xenoestrogens Zearalenone and Bisphenol A in Human Ovarian Cells

Éva Márton, Alexandra Varga, András Penyige, Zsuzsanna Birkó, István Balogh, Bálint Nagy and Melinda Szilágyi

Table S9. Primer sequences of 18 genes applied in the qPCR experiments.

	Forward	Reverse
RBBP8NL	CGCATCTTCATCCTCACCA	CCTCCTTCAAGGTCTCGTTCT
MYC	TGCTCCATGAGGAGACACC	CTTTTCCACAGAAACAACATCG
EGR1	AGCCCTACGAGCACCTGAC	GGTTTGGCTGGGGTAACTG
NOLC1	AAGACAGCAGTGATGAGTCTGATT	AGCTCTCTGCTGCTTTCTTTG
RRP12	ACCATCCGGCCCTACCTA	CCTGAGGACTGGCACACA
BLNK	AGAGAGCCCTGCTGACGA	CGAGTGCTCATCTGGATTTTC
TGM1	GGACTCAGTACTGCGGTTGC	TGGAGATGGCGTGGTAGG
KRT4	TTTGCCTCCTTCATCGACA	TCAAGGTTTTTGCTGGAGGT
BMF	GAGACTCTCTCCTGGAGTCACC	CCTCTGGTTGGAACACATCA
CD24	ATGGGCAGAGCAATGGTG	CCAGTTGTTGTTTCACTGGAAT
NOTCH3	CTTCCTGCGATCAGGACATC	CACTCATCCACATCGCGGG
GBP3	CCAGCGATCCAGCGAAAGAA	GGGCCTGTCATGTGGATCTC
MYBL1	AGGATCGGGGAGAAGGAGCAT	TCGGCATACTGAAGGTCATCATC
GREB1	TCTTGCACAATTCCATCGAG	GTCCACTCGGCTACCACCT
CA12	GTTTCTCCTGACCAACAATGG	CGTGGCACTGTAGCGAGAC
DEPTOR	TCATGGCATCTGAATTCCTG	ATGGGTGCTTGTTGGACAC
AGT	TGCCGTTGTTCTGGGTACTA	TGTATGTACACCCGGTCACCT
GAPDH	CACCCACTCCTCCACCTTT	GCCAAATTCGTTGTCATACCA
